# The complete mitogenome of *Liniparhomaloptera qiongzhongensis* (Cypriniformes: Gastromyzontidae) and phylogenetic implications

**DOI:** 10.1080/23802359.2021.1955770

**Published:** 2021-07-22

**Authors:** Shuqing Deng, Xingwei Cai, Siyu Yuan, Unisa Conteh Kanu, Ke Jin, Zhixin Shen

**Affiliations:** a The Changjiang Civilization Museum (Wuhan Natural History Museum), Wuhan, China; b Institute of Hydrobiology, Chinese Academy of Sciences, Wuhan, China; c Hainan Academy of Ocean and Fisheries Sciences, Haikou, China; d Fisheries College, Huazhong Agricultural University, Wuhan, China; e Hubei Institute of Geosciences, Wuhan, China

**Keywords:** *Liniparhomaloptera qiongzhongensis*, mitochondrial genome, taxonomy, phylogenetic relationship

## Abstract

*Liniparhomaloptera qiongzhongensis* Zheng and Chen 1980 is distributed in Hainan Island, China. The complete mitogenome of the species was sequenced in this study. It had a circular molecule of 16,554 bp in length with a total of 56.5% A + T content, containing 13 protein-coding genes (PCGs), 22 tRNA genes, two rRNA genes, and one control region. Five overlapping regions and 14 intergenic sequences were observed with the length of 2–10 bp and 1–31 bp, respectively. The phylogenetic analysis based on 13 protein-coding genes revealed that *L. qiongzhongensis* had the closest relationship with *L. disparis*. This study would provide useful genetic information for future studies on taxonomy, phylogeny and evolution of *Liniparhomaloptera* species.

Lin (1934) described *Parhomaloptera dispairs* using samples from the Peal River, Guangdong, China. Fang (1935) later established *Liniparhomaloptera* (Gastromyzontidae) as a new genus to accommodate *Parhomaloptera dispairs.* Zheng and Chen (1980) treated the specimens named *Liniparhomaloptera disparis* in the Peal River as *L. disparis disparis*, and described a new subspecies, *L. disparis qiongzhongensis* based on the materials from Wanquan-He, Hainan Province. Given that the two subspecies had isolated distributions, they were treated as distinct species by Kottelat (2012). Subsequent research based on morphological method found they had significant differences in infralabial shape and head spot pattern (Yang et al. 2014). In this study, molecular method was used to investigate the phylogenetic relationships of *Liniparhomaloptera*, which would provide useful basis for future studies on taxonomy, phylogeny and evolution of the genus. We firstly determined the complete mitochondrial genome of *L. qiongzhongensis*.

The specimen was collected from Lingshuihe River (N 18°44′45.8″, E 109°44′32.3″), Hainan Province, China, in December 2019, and stored in the Museum of Aquatic Organisms at the Institute of Hydrobiology (IHB), Chinese Academy of Sciences (voucher specimen: IHB2019120242). Genomic DNA was extracted from the pelvic fin. Sequences were successfully amplified by PCR (polymerase chain reaction) with 16 pairs of primers (Supplementary Table S1), and PCR was conducted using the following conditions: denaturation at 95 °C for 2 min, 35 amplification cycles (94 °C denaturation for 10 s, 55–60 °C annealing for 30 s, 72 °C extension for 60–90 s), and a final extension at 72 °C for 5 min. Amplified products were subsequently purified and sequenced by a commercial sequencing company using Sanger sequencing technology. The mitochondrial genome was annotated through MitoAnnotator (Iwasaki et al. 2013). After using the BLAST analysis in Genbank, we submitted the genome into GenBank with accession number MZ047229.

The mitochondrial genome of *L. qiongzhongensis* was a closed double-stranded circular molecule of 16,554 bp It contained 13 protein-coding genes (ATP6, ATP8, COI–III, Cytb, ND1–6, ND4L), 22 tRNA genes, two rRNA genes (12S and 16S rRNA), and one control region (D-Loop). The base composition was 30.1% of A, 16.4% of G, 26.4% of T, 27.1% of C, with the A + T contents of 56.5%. The ND6 gene and eight tRNA genes were encoded on the L-strand; the remaining mitochondrial genes located on the H-strand. There were five overlapping regions, varying from 2 to 10 bp in length. 14 intergenic sequences were observed with 1–31bp in length. The total length of the PCGs (13 protein-coding genes) was 11,428 bp, and these genes encode 3800 amino acids. The 22 tRNA genes had lengths ranging from 66 bp (tRNACys) to 76 bp (tRNALys). The 12S and 16S rRNA genes were 955 and 1673 bp, respectively. D-loop was 895 bp in length.

The newly sequenced and other 13 species of Gastromyzontidae were chosen as the ingroup, and *Lepturichthys fimbriata* and *Jinshaia sinensis* selected as the outgroup. The concatenated protein-coding gene sequences were extracted by PhyloSuite (Zhang et al. 2020). The phylogenetic trees were inferred using Bayesian inference (BI) and maximum likelihood (ML) approaches to determine the phylogenetic position of *Liniparhomaloptera* within Gastromyzontidae. Bayesian and ML analyses were conducted using MrBayes (Ronquist et al. 2012) and IQ-TREE (Nguyen et al. 2015) based on 13 PCGs. The genetic distances (p-distance with 1000 bootstraps) were calculated by MEGA 7.0 (Kumar et al. 2016). Both methods (BI and ML) showed an identical topology (Figure 1). From the tree topology, *L. qiongzhongensis* had a closer relationship with *L. disparis* than with other species. The nucleotide sequence divergence of 13 PCGs between the two species was 11.3%, larger than the genetic distances (2.1%) between *Lepturichthys fimbriata* and *Jinshaia sinensis*. The results obtained herein would provide an important basis for further studies on taxonomy, phylogeny, and evolution of *Liniparhomaloptera* species.

**Figure 1.**
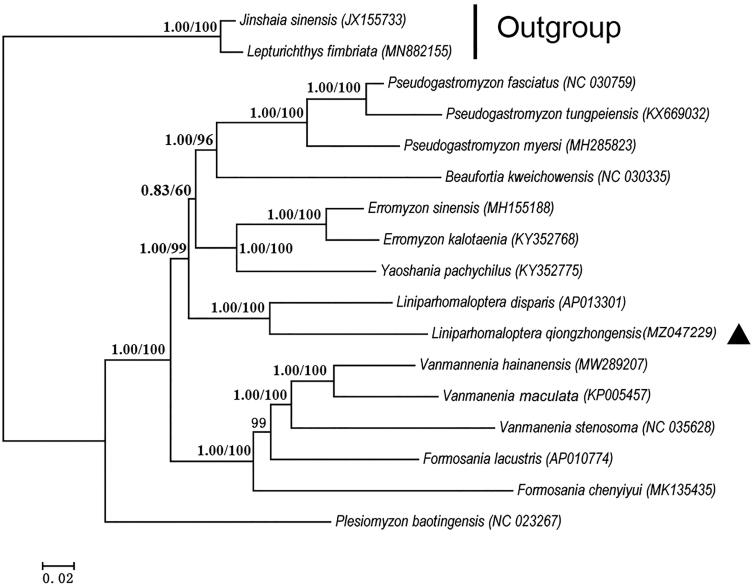
Phylogenetic tree of *Liniparhomaloptera* species based on 13 concatenated protein-coding genes. The left and right numbers at each node represent Bayesian posterior probability and maximum likelihood bootstrap value, respectively. The GenBank accession number of each species was shown in brackets. Solid triangle indicates the newly sequenced mitogenome.

## Data Availability

The genome sequence data that support the findings of this study are openly available in GenBank of NCBI at [https://www.ncbi.nlm.nih.gov/nuccore/MZ047229] (https://www.ncbi.nlm.nih.gov/nuccore/MZ047229) under the accession no. MZ047229.
